# Commentary Letter to the Editor Regarding Impact of Intrauterine Infusion of Autologous Platelet‐Rich Plasma on Assisted Reproductive Outcomes in Patients With Recurrent Implantation Failure: A Meta‐Analysis of Randomized Controlled Trials

**DOI:** 10.1002/rmb2.70091

**Published:** 2026-08-18

**Authors:** Germar‐Michael Pinggera, Bettina Toth, Giovanni Maria Colpi

**Affiliations:** ^1^ Department of Urology Medical University of Innsbruck Innsbruck Austria; ^2^ Gynecological Endocrinology and Reproductive Medicine Medical University of Innsbruck Innsbruck Austria; ^3^ Andrology and IVF Center Next Fertility Procrea Lugano Switzerland

## Abstract

We commend Liang et al. for their rigorous meta‐analysis of intrauterine platelet‐rich plasma infusion in ART, particularly for recurrent implantation failure, but note issues affecting interpretation. Forest plot axes label “favors control/PRP” using a harm‐oriented convention, inverting standard visual interpretation. The methods state a random‐effects model, yet reported effect sizes and heterogeneity statistics match fixed‐effect estimation, likely narrowing confidence intervals and overstating precision for live birth rate. One figure omits two included studies without explanation, undermining transparency. Non‐significant subgroup findings are described as clinically meaningful, risking overinterpretation absent pre‐specified hypotheses or multiplicity correction. Accurate, outcome‐specific labeling and internally consistent model reporting are essential safeguards against misreading otherwise sound evidence, given its direct bearing on patient counseling and clinical decisions. We encourage a clarifying erratum to preserve this work's scientific integrity and clinical utility.

Dear Editor,

We read the recent meta‐analysis by Liang et al. on intrauterine autologous platelet‐rich plasma (PRP) infusion and its possible effects on medical assisted reproduction among patients with recurrent implantation failure (RIF) with considerable interest [1]. The authors are to be commended for a methodologically sound study that adds meaningful evidence to the reproductive medicine literature and corroborates earlier findings in this area [2, 3].

The study has several strengths worth highlighting. Restricting inclusion to randomized controlled trials and incorporating the most recent published evidence (10 RCTs, *n* = 1188) strengthens the level of evidence relative to earlier reviews that combined observational and randomized designs. The pooled estimate for clinical pregnancy rate (RR 2.23, 95% CI 1.84–2.69) with no observed heterogeneity (*I* ^2^ = 0%) is notable, and the concordant direction of effect across implantation rate, ongoing pregnancy rate, and live birth rate (LBR) (RR 3.74, 95% CI 2.68–5.22) supports a genuine treatment effect [1]. The leave‐one‐out sensitivity analysis identifying the Nazari et al. 2022 trial as the principal source of heterogeneity in the LBR, and the subgroup analyses on PRP dose (0.5 mL vs. 0.5–1.0 mL) and embryo transfer stage (blastocyst vs. cleavage), add useful exploratory detail even though neither subgroup interaction reached statistical significance.

We would, however, like to raise several points that, in our view, deserve further comment or clarification.

1. Forest plots (Figures 3–11): all figures use the axis annotation “Favors experimental” on the left of the null line (RR < 1) and “Favors control” on the right (RR > 1). This convention is appropriate for outcomes representing harm, where RR > 1 signals increased risk in the experimental arm and therefore favors the control group. For the outcomes examined here—clinical pregnancy, live birth, implantation, and ongoing pregnancy rates—the event measured is a beneficial outcome. In this context, RR > 1 indicates that the experimental (PRP) group experienced the favorable event more often. The direction of interpretation is therefore reversed relative to what the current axis labels suggest. This labeling convention, while historically common in dichotomous outcome forest plots, is no longer recommended by the Cochrane Handbook (Higgins et al.), which explicitly states that this default convention is no longer encouraged since it is not universally appropriate, [4] and instead calls for transparent, outcome‐specific axis labeling rather than defaulting to the ‘favors experimental = left’ convention originally designed for unfavorable/harm‐related outcomes [4, 5, 6]. This is not a numerical or statistical error—the pooled RR of 2.23 and its 95% CI are correctly calculated and interpreted as evidence favoring PRP. Rather, it is a labeling convention issue inherited from default forest‐plot software templates (e.g., RevMan), which typically assume a harmful outcome unless manually adjusted. We respectfully suggest that the axis labels should be requested by the editor and reviewers reading “Favors control” on the left and “Favors experimental” on the right for the beneficial outcomes (CPR, LBR, IR, OPR, chemical pregnancy rate), while keeping the present orientation for the miscarriage rate figure, where a lower RR does favor the experimental group. Such precise axis labeling would prevent potential misinterpretation by readers who scan figures without cross‐referencing the accompanying text, and would further enhance the manuscript's already high standard of transparency.
2. Furthermore, while the Methods explicitly specify use of the DerSimonian‐Laird random‐effects model to pool effect sizes was defined, every forest plot presented (Figures 3–11) is labeled “M‐H, Fixed, 95% CI”—indicating that Mantel–Haenszel fixed‐effect pooling was actually used throughout the results, not the stated random‐effects model. This discrepancy is not trivial: for the live birth rate outcome, where *I* ^2^ = 79% (substantial heterogeneity), a fixed‐effect model inappropriately narrows the confidence interval and overstates precision, potentially producing a false impression of a stronger, more certain effect (RR 3.74, 95% CI 2.68–5.22) than a random‐effects model would yield [7].
3. As a minor comment for Figure 4 we observed a discrepancy between the text stating “All 10 studies compared the chemical pregnancy rate between the two groups” (518 vs. 521 participants), but the corresponding figure lists only 8 studies, omitting Fazaeli 2024 and Zargar 2021 without explanation. This should be considered a data‐reporting inconsistency between narrative text and the actual pooled figure.
  Typical for meta‐analysis is the inclusion of biologically distinct variables such as different PRP preparation protocols across the included studies. In the subgroup analysis the different PRP dose (0.5 mL vs. 0.5–1.0 mL) showed no significant differences with a *p* = 0.34, *I* ^2^ = 0%. Nevertheless the authors described in the abstract and the discussion section these as clinically meaningful (“low‐dose PRP… seems to be more effective,” “notably greater treatment effect… in blastocyst transfer cycles”). This amounts to overreach, since non‐significant, exploratory subgroup comparisons are being presented as if they were near‐conclusive clinical guidance.
4. The included trials (*n* = 10) all originate from a single country (Iran), a fact stated in Table 1 but not discussed as a limitation within the discussion; the authors should clarify whether a language or geography‐based restricted sensitivity analysis was considered, and justify the English‐only search restriction given this striking single‐country concentration of evidence. This single‐country concentration may introduce systemic confounding through shared healthcare practices, population genetics, PRP protocols, and overlapping research teams. Given that endometrial receptivity, baseline platelet biology, and standard IVF protocols can vary across populations, we think this geographic homogeneity deserves explicit mention as a constraint on the generalizability of the pooled estimates to other ethnic or healthcare settings. The absence of heterogeneity (*I* ^2^ = 0%) for the primary outcome may in part reflect this homogeneity in study population and protocol rather than a universally consistent treatment effect, and we would welcome the authors' view on this point.
5. The risk‐of‐bias assessment (Figure 2) shows high risk of performance and detection bias across most trials, attributable to the inherent difficulty of blinding an intrauterine infusion procedure. This is acknowledged in the manuscript through the moderate GRADE‐type downgrade, but its practical implication—namely that patients' or clinicians' awareness of treatment allocation could influence outcome ascertainment, particularly for subjectively timed outcomes—is not discussed further. We think a brief comment on how this might have affected the magnitude of the pooled clinical pregnancy and live birth estimates would be useful.
6. We noticed that implantation and ongoing pregnancy rate were each derived from only three and four trials, respectively, and that the definition of RIF itself varied across the included studies (three versus more than three prior failed transfers). The sensitivity analysis by RIF definition showed a smaller and borderline‐significant effect in the stricter subgroup (RR 1.56, 95% CI 0.98–2.48, *p* = 0.06), which is an important nuance that could be given more emphasis in the discussion, since it suggests that the magnitude of benefit may be smaller in the most severely affected patients.
  Finally, building on the findings of this study, endometrial‐focused interventions such as PRP infusion address only one side of the implantation process. Since sperm quality and selection also influence embryo developmental competence, we suggest that, alongside appropriate counseling of the male partner, [8] future prospective trials examine whether combining PRP‐based endometrial preparation with advanced sperm selection methods e.g., microfluidic sperm sorting, surface‐charge selection (Zeta potential or electrophoresis), apoptosis‐based selection through magnetic‐activated cell sorting, or hyaluronic acid‐binding assays, and high‐magnification morphology selection, applied singly or in combination–might further raise clinical pregnancy rates and, more importantly, LBR in couples with RIF. Current evidence on these techniques, whether used alone or combined, remains inconsistent for live birth as an endpoint, [9, 10, 11, 12] and this inconsistency is itself a reason to pursue combined endometrial and gamete‐focused strategies in future trial design rather than treating the two sides of implantation separately.

We again commend the authors for this valuable and carefully conducted meta‐analysis and look forward to future work building on these important findings.

## Conflicts of Interest

The authors declare no conflicts of interest.

## Data Availability

The data that support the findings of this study are available on request from the corresponding author. The data are not publicly available due to privacy or ethical restrictions.
